# Temporal correlation coefficient for directed networks

**DOI:** 10.1186/s40064-016-2875-0

**Published:** 2016-07-28

**Authors:** Kathrin Büttner, Jennifer Salau, Joachim Krieter

**Affiliations:** Institute of Animal Breeding and Husbandry, Christian-Albrechts-University, Olshausenstr. 40, 24098 Kiel, Germany

**Keywords:** Temporal network, Temporal correlation coefficient, Directed network, Topological overlap

## Abstract

Previous studies dealing with network theory focused mainly on the static aggregation of edges over specific time window lengths. Thus, most of the dynamic information gets lost. To assess the quality of such a static aggregation the temporal correlation coefficient can be calculated. It measures the overall possibility for an edge to persist between two consecutive snapshots. Up to now, this measure is only defined for undirected networks. Therefore, we introduce the adaption of the temporal correlation coefficient to directed networks. This new methodology enables the distinction between ingoing and outgoing edges. Besides a small example network presenting the single calculation steps, we also calculated the proposed measurements for a real pig trade network to emphasize the importance of considering the edge direction. The farm types at the beginning of the pork supply chain showed clearly higher values for the outgoing temporal correlation coefficient compared to the farm types at the end of the pork supply chain. These farm types showed higher values for the ingoing temporal correlation coefficient. The temporal correlation coefficient is a valuable tool to understand the structural dynamics of these systems, as it assesses the consistency of the edge configuration. The adaption of this measure for directed networks may help to preserve meaningful additional information about the investigated network that might get lost if the edge directions are ignored.

**Electronic supplementary material:**

The online version of this article (doi:10.1186/s40064-016-2875-0) contains supplementary material, which is available to authorized users.

## Background

Network theory has become a valuable framework in many different research areas, whenever the system under investigation can be described as a graph, thus a set of nodes and edges connecting theses nodes. For instance, social contacts of individuals (Kasper and Voelkl 2009; Krause et al. 2007; Lewis et al. 2008; Makagon et al. 2012), disease transmission (Eames and Read 2008; Eubank 2005; Heckathorn et al. 1999), trade networks (Büttner et al. 2013, 2015; Guimerà et al. 2005; Kaluza et al. 2010; Nöremark et al. 2011), the World Wide Web and the internet (Albert et al. 1999; Barabási et al. 2000; Cohen et al. 2000) or citation networks (Newman 2001a, b), to name but a few.

These previous studies dealing with network analysis focused mainly on static network analysis, meaning an edge was drawn between a pair of nodes whenever there was a contact between these two nodes during the whole observation period.

A static network *G* = (*V*, *E*), where *V* is the set of nodes and *E* is the set of edges, can be illustrated as so-called adjacency matrix (*a*_*ij*_)_*ij*_ with *a*_*ij*_ = 1 if there is an edge between nodes *i* and *j*, and *a*_*ij*_ = 0 otherwise. Thus, the temporal information is neglected. However, in order to avoid losing all temporal information of the system, one possible approach is to separate the observation window in smaller parts and aggregate the contacts only over the formed snapshots, which are then analysed separately (examples are Bajardi et al. 2011; Büttner et al. 2015; Dubé et al. 2011; Nöremark et al. 2011; Rautureau et al. 2012; Vernon and Keeling 2009). Therefore, not all temporal information gets lost.

Although the static network analysis neglects partly the temporal variation in the system under investigation, one of its big advantages lies in the huge toolbox of methods that has been developed in the last decades. They range from network parameters describing the whole network topology (e.g. diameter, degree distribution, density, fragmentation index) to centrality parameters which allow for node ranking (e.g. in- and out-degree, closeness centrality, betweenness centrality) (Newman 2010; Wasserman and Faust 1994).

Depending on the system under investigation, the static network analysis can capture its temporal dynamics sufficiently well, but if the temporal variation in the system is too high the static network analysis is unable to display this variation. This leads us to the so-called temporal networks (Holme and Saramäki 2012; Kempe et al. 2002). However, the analysis of temporal networks is an interdisciplinary field which is still in its infancy and therefore, the analytical and computational methods are still at an early stage of development (Masuda and Holme 2013).

Therefore, to assess the quality of the static aggregation compared to the temporal counterpart some measures have been developed. For example, the causal fidelity measures the fraction of the number of paths in a static network, which can also be taken in the temporal counterpart (Lentz et al. 2013). Another example is the temporal correlation coefficient. It is a measure of the overall average probability for an edge to persist across two consecutive snapshots and can be used for the quality assessment of the static aggregation (Nicosia et al. 2013; Tang et al. 2010). In the first case, the measure can be used for both undirected and directed networks, i.e. the edge direction is taken into account resulting in an asymmetric adjacency matrix. However, up to now, the temporal correlation coefficient is only defined for undirected networks. Taking the edge direction into account is of special importance for many research questions. For example, in behavioural sciences, it is important to know which individual is the active part and which is the passive part of an interaction or, in trade networks, which node is the supplier and which is the purchaser. Thus, working with directed networks provides more information and reveals further insights into the interaction between the individual nodes.

Therefore, we introduce in the present paper an adaption of the temporal correlation coefficient first presented by Tang et al. (2010) and Nicosia et al. (2013), that was modified by Pigott and Herrera (2014) and Büttner et al. (2016), to directed networks.

## Methods

The first part of the materials and methods section deals with the temporal correlation coefficient for undirected networks. Here, a detailed description of the individual calculation steps is entailed. In the second paragraph, the adaption of this measure for directed networks is presented. In the last paragraph of the materials and methods section, both the undirected and the directed calculation of the temporal correlation coefficient are carried out on a real-word pig trade network of a producer community in Northern Germany. This comparison clarifies the importance of a distinction between the undirected and the directed temporal correlation coefficient.

### Temporal correlation coefficient

The temporal correlation coefficient *C* measures the overall average probability for an edge to persist across two consecutive snapshots (Nicosia et al. 2013; Tang et al. 2010). The calculation of *C* is divided into three individual calculation steps.

Firstly, for all nodes *i* = 1, …, *N*, where *N* is the total number of nodes in the network, and all snapshots *t*_*m*_, with *m* = 1, …, *M* − 1, where *M* is the total number of considered snapshots, the topological overlap *C*_*i*_(*t*_*m*_, *t*_*m*+1_) of node *i* between two consecutive snapshots *t*_*m*_ and *t*_*m*+1_ is calculated with the following formula:

1 $$C_{i} \left( {t_{m} , t_{m + 1} } \right) = \frac{{\mathop \sum \nolimits_{j} a_{ij} \left( {t_{m} } \right)a_{ij} \left( {t_{m + 1} } \right)}}{{\sqrt {\left[ {\mathop \sum \nolimits_{j} a_{ij} (t_{m} )} \right]\left[ {\mathop \sum \nolimits_{j} a_{ij} \left( {t_{m + 1} } \right)} \right]} }},$$

where *a*_*ij*_ denotes an entry in the unweighted adjacency matrix of the graph. Therefore, summing over *a*_*ij*_ illustrates the interaction between *i* and every other node for the two consecutive snapshots *t*_*m*_ and *t*_*m*+1_.

Secondly, the average topological overlap of the graph *C*_*m*_ for two consecutive snapshots *t*_*m*_ and *t*_*m*+1_ is determined. According to the proposed adaption of the temporal correlation coefficient by Büttner et al. (2016), *C*_*m*_ is calculated as follows,

2 $$C_{m} = \frac{1}{{\hbox{max} \left[ {A\left( {t_{m} } \right), A\left( {t_{m + 1} } \right)} \right]}}\mathop \sum \limits_{i = 1}^{N} C_{i} \left( {t_{m} , t_{m + 1} } \right) ,$$

where *max*[*A*(*t*_*m*_), *A*(*t*_*m*+1_)] denotes the maximum number of active nodes of the graph at *t*_*m*_ and *t*_*m*+1_. A node *i* is called “active” at time *t*_*m*_, if there exists a node *j* ≠ *i* and an edge between *i* and *j* in the graph at *t*_*m*_, i.e. node *i* has a degree greater than zero.

Despite the calculation of *C*_*m*_, the average topological overlap of the nodes *C*_*i*_ for all snapshots can be calculated as follows,

3 $$C_{i} = \frac{1}{M - 1}\mathop \sum \limits_{m = 1}^{M - 1} C_{i} \left( {t_{m} , t_{m + 1} } \right).$$

In the third calculation step, summing up all results for the topological overlap gives the temporal correlation coefficient *C* of the network:

4 $$C = \frac{1}{M - 1}\mathop \sum \limits_{m = 1}^{M - 1} C_{m}$$

The range of the values of all three calculation steps is between 0 and 1. One indicates that in the observed snapshots the edge configuration is identical, whereas zero means that none of the same edges are common in the observed snapshots.

**Fig. 1:**
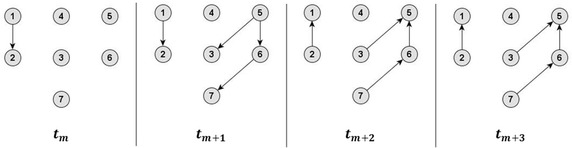
Example network of 4 different temporal snapshots (*t*_*m*_, …, *t*_*m*+3_)

Figure 1 depicts an example network consisting of 4 temporal snapshots with directional information given by the arrow tips of the edges. In the Additional file 1 the single calculation steps of the undirected temporal correlation coefficient *C* are explained in detail.

### Adaption of the temporal correlation coefficient to directed networks

**Fig. 2:**
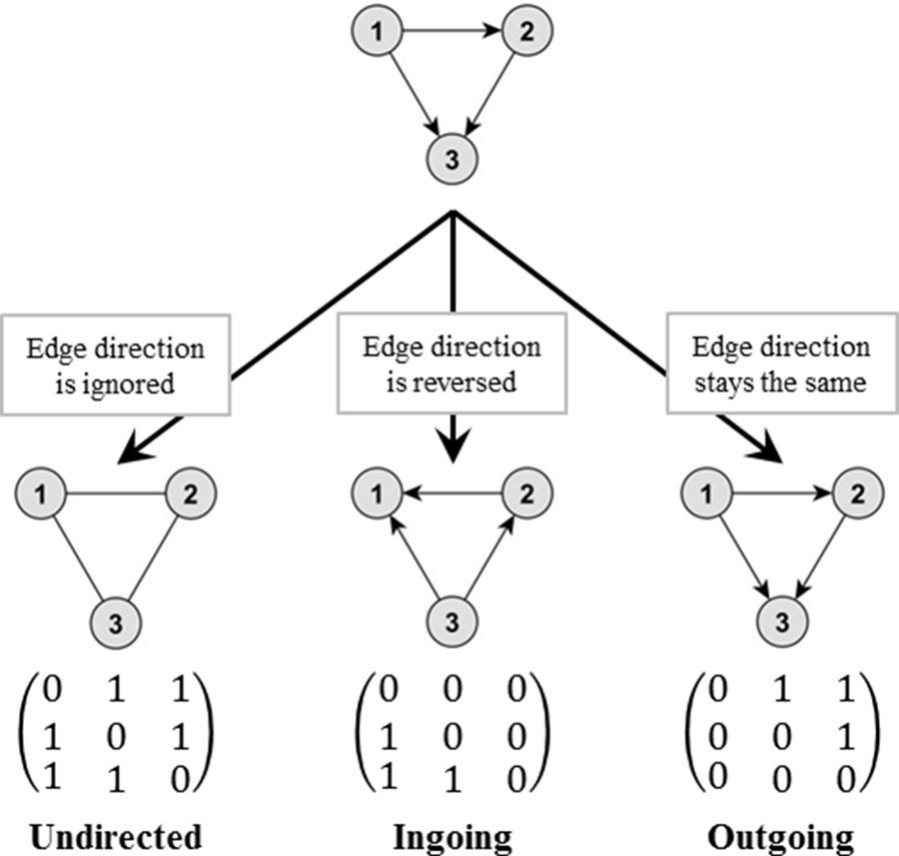
Differences between the undirected and directed (ingoing and outgoing) representation of an example network. In the undirected case, the edge direction is ignored. In the ingoing case, the edge direction is reversed, meaning the adjacency matrix is transposed, and the ingoing edges are addressed instead of the outgoing edges. In the outgoing case, the original adjacency matrix is used addressing the outgoing edges

In directed networks a distinction between ingoing and outgoing edges is made. In undirected networks an edge from node *i* to node *j*—corresponding to *a*_*ij*_ = 1—is additionally considered as an edge from node *j* to node *i*, which implies *a*_*ji*_ = 1. Therefore, the adjacency matrices of undirected networks are symmetrical. This is no longer the case for directed networks, as there could be an edge from node *i* to node *j*, although no edge from node *j* to node *i* exists, which implies $1 = a_{ij} \ne a_{ji} = 0$. Figure 2 shows an example for the differences between the undirected and the directed representation of a network with regard to its adjacency matrices.

Therefore, the temporal correlation coefficient in directed networks should be calculated for the configuration of the ingoing edges (hereinafter named as ingoing temporal correlation coefficient *C*^*in*^) and for the configuration of the outgoing edges (hereinafter named as outgoing temporal correlation coefficient *C*^*out*^). Due to the fact that the maximum number of active nodes max[*A*(*t*_*m*_), *A*(*t*_*m*+1_)] is used to calculate the temporal correlation coefficient in undirected networks, where *A*(*t*_*m*_) is the number of nodes with nonzero degree in the snapshot *t*_*m*_, this has to be adapted while dealing with directed networks. In the calculation of *C*^*in*^ and *C*^*out*^, *A*(*t*_*m*_) will be replaced by the number of nodes with nonzero in-degree *A*^*in*^(*t*_*m*_) and the number of nodes with nonzero out-degree *A*^*out*^(*t*_*m*_), respectively.

#### Ingoing temporal correlation coefficient *C*^*in*^

For the calculation of the ingoing temporal correlation coefficient *C*^*in*^, Eq. (1) is used with the transposed adjacency matrix to focus on the ingoing edges (Fig. 2). The values $C_{i}^{in} \left( {t_{m} , t_{m + 1} } \right)$ from this calculation step are then used in Eq. (5). In contrast to Eq. (2), instead of *max*[*A*(*t*_*m*_), *A*(*t*_*m*+1_)], *max*[*A*^*in*^(*t*_*m*_), *A*^*in*^(*t*_*m*+1_)] is used for the calculation of $C_{m}^{in}$:

5 $$C_{m}^{in} = \frac{1}{{\hbox{max} \left[ {A^{in} \left( {t_{m} } \right), A^{in} \left( {t_{m + 1} } \right)} \right]}}\mathop \sum \limits_{i = 1}^{N} C_{i}^{in} \left( {t_{m} , t_{m + 1} } \right)$$

In addition to $C_{m}^{in}$ and similarly to Eq. (3), the average topological overlap of the nodes for all possible snapshots can also be calculated for the ingoing edges $C_{i}^{in}$ and is calculated as follows,

6 $$C_{i}^{in} = \frac{1}{M - 1}\mathop \sum \limits_{m = 1}^{M - 1} C_{i}^{in} \left( {t_{m} , t_{m + 1} } \right)$$

For the last calculation step no changes in Eq. (4) are carried out.

A detailed description of the single calculation steps for the ingoing temporal correlation coefficient *C*^*in*^ for the example network (Fig. 1) is illustrated in the Additional file 1.

#### Outgoing temporal correlation coefficient *C*^*out*^

For the calculation of the outgoing temporal correlation coefficient, the first calculation step (Eq. 1) stays the same, as the outgoing edges are represented by the untransposed adjacency matrix (Fig. 2). The values $C_{i}^{out} \left( {t_{m} , t_{m + 1} } \right)$ are then used in Eq. (7). Here, $max\left[ {A^{out} \left( {t_{m} } \right), A^{out} \left( {t_{m + 1} } \right)} \right]$ is used for the calculation of $C_{m}^{out}$:

7 $$C_{m}^{out} = \frac{1}{{\hbox{max} \left[ {A^{out} \left( {t_{m} } \right), A^{out} \left( {t_{m + 1} } \right)} \right]}}\mathop \sum \limits_{i = 1}^{N} C_{i}^{out} \left( {t_{m} , t_{m + 1} } \right)$$

Additionally, the average topological overlap of the nodes for all possible snapshots for the outgoing case $C_{i}^{out}$ can be calculated as follows,

8 $$C_{i}^{out} = \frac{1}{M - 1}\mathop \sum \limits_{m = 1}^{M - 1} C_{i}^{out} \left( {t_{m} , t_{m + 1} } \right)$$

For the last calculation step no changes in Eq. (4) are carried out.

A detailed description of the single calculation steps for the outgoing temporal correlation coefficient *C*^*out*^ for the example network (Fig. 1) is illustrated in the Additional file 1.

#### Convergence behaviour of the temporal correlation coefficient (*C*, *C*^*in*^ and *C*^*out*^)

As the topological overlap is a probability, it is expected that for all *m* = 1, …, *M* − 1 the average topological overlap *C*_*m*_ in the undirected case, as well as $C_{m}^{in}$ and $C_{m}^{out}$ for the ingoing and outgoing case, respectively, are in the range of 0–1. The values for *C*_*m*_ between two consecutive snapshots equal 1, only if these snapshots show identical edge configuration. To reveal a convergence behaviour of the temporal correlation coefficients (*C*, *C*^*in*^ and *C*^*out*^), an identical extension of the time series was generated by attaching the last snapshot, i.e. the graph at *t*_*M*_ of the example network of Fig. 1, repeatedly to the existing dynamic network until the length of this series of networks equalled 100. In Büttner et al. (2016) it has already been illustrated that the corresponding series of undirected temporal correlation coefficients *C*, calculated with the method proposed there, converges towards 1. The series of directed ingoing and outgoing temporal correlation coefficients corresponding to the series of identical extensions of the example network of Fig. 1 were calculated, and their convergence behaviour was plotted and analysed.

### Temporal correlation coefficient (*C*, *C*^*in*^ and *C*^*out*^) calculated for a pig trade network

#### Data basis

From June 2006 to May 2009, pig movement data from a producer community in Northern Germany were recorded. This corresponds to an observation window length of 1096 days. The data contained the date of the movement, the supplier, the purchaser as well as the batch size and the type and age group of the delivered animals. In total, the data comprised 4635 animal movements between 483 farms which could be categorized in 29 multipliers, 34 farrowing farms, 153 finishing farms and 267 farrow-to-finishing farms. Due to the dead-end characteristic of the abattoirs they were excluded from the network analysis.

#### Network construction

In this pig trade network, the farms illustrate the nodes of the network and the trade contacts between the farms represent the edges. Networks with increasing time window lengths (1–548 days) were constructed in order to evaluate how the chosen time window length may affect the outcome of the temporal correlation coefficient. This means that for a time window length of 1 day 1096 single snapshots of the network were created. If the time window length is doubled to 2 days, the number of snapshots halves to 548. And finally for a time window length of 548 days, only 2 single snapshots were generated. Due to the predefined length of the observation window with 1096 days, the last snapshot may contain less days than the previous snapshots. This was the case if the length of the time window was not a proper divisor of 1096 which corresponds to the length of the whole observation period. These incomplete snapshots were excluded from the further analysis.

### Frequency distributions

In order to get more information about the frequency distribution of the average topological overlap of the nodes (*C*_*i*_, $C_{i}^{in}$ and $C_{i}^{out}$) the values were categorized for each time window length and each farm type into the following 6 categories: 0: *C*_*i*_ = 0, 1: 0 < *C*_*i*_ ≤ 0.2, 2: 0.2 < *C*_*i*_ ≤ 0.4, 3: 0.4 < *C*_*i*_ ≤ 0.6, 4: 0.6 < *C*_*i*_ ≤ 0.8, 5: 0.8 < *C*_*i*_ ≤ 1. Then the percentage of each category was calculated and plotted as a grouped bar chart.

### Statistical analysis

All statistical analyses were performed using SAS^®^ statistical software package (SAS^®^ Inst. Inc. 2008). The adaption of the temporal correlation coefficient was carried out with the programming language Python (van Rossum and Drake 2001).

## Results

### Convergence behaviour of the undirected, ingoing and outgoing temporal correlation coefficient (*C*, *C*^*in*^ and *C*^*out*^)

**Fig. 3:**
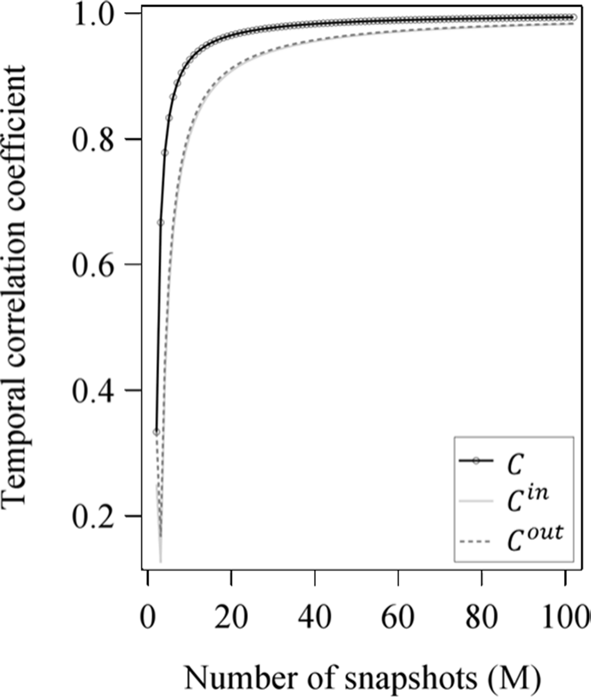
Convergence behaviour of the temporal correlation coefficient. Convergence behaviour of the undirected, ingoing and outgoing temporal correlation coefficient (*C*, *C* ^*in*^ and *C* ^*out*^) depending on the increasing number of identical snapshots added to the series of the example network of Fig. 1

Figure 3 shows the convergence behaviour of the temporal correlation coefficient for the undirected and the directed (ingoing and outgoing) calculation for the example network of Fig. 1 depending on the increasing number of added identical snapshots. Both *C*^*in*^ and *C*^*out*^ showed lower values than *C*. However, with increasing number of snapshots added to the time series, all three possible calculations converged to 1 from below.

### Temporal correlation coefficient (*C*, *C*^*in*^ and *C*^*out*^) calculated for a pig trade network

**Fig. 4:**
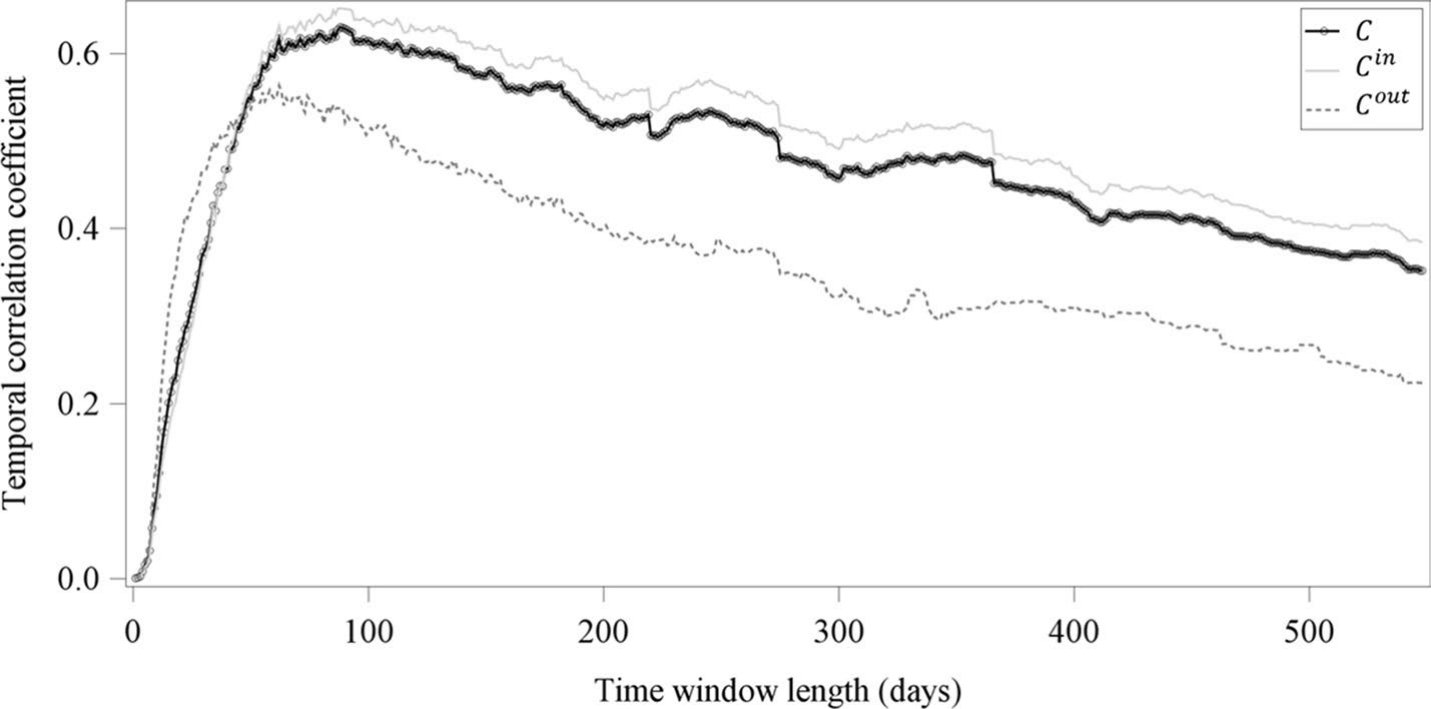
Course of the temporal correlation coefficient for different time window lengths. Undirected, ingoing and outgoing temporal correlation coefficient (*C*, *C* ^*in*^ and *C* ^*out*^) of the pork supply chain of a producer community in Northern Germany for different time windows lengths

For the pork supply chain under investigation, the highest mean value over all possible time window lengths (n = 548) could be obtained for *C*^*in*^ with 0.50 ± 0.11, followed by *C* with 0.47 ± 0.11. The lowest mean value showed *C*^*out*^ with 0.36 ± 0.10. These results could be confirmed by Fig. 4, where *C* and *C*^*in*^ showed a quite similar course, whereas *C*^*out*^ had clearly lower values. Despite this, all three measures increased rapidly from close to 0 to their maximum values from the time window of length 1 to a window length of 62 days for *C*^*out*^ (max = 0.56) and 88 days for both *C* (max = 0.63) and *C*^*in*^ (max = 0.65). After reaching their maximum, the values decreased slowly with increasing length of the time window to 0.38 for *C*^*in*^, 0.35 for *C* and 0.22 for *C*^*out*^.

### Average topological overlap of the nodes (*C*_*i*_, $C_{i}^{in}$ and $C_{i}^{out}$) calculated for a pig trade network

**Fig. 5:**
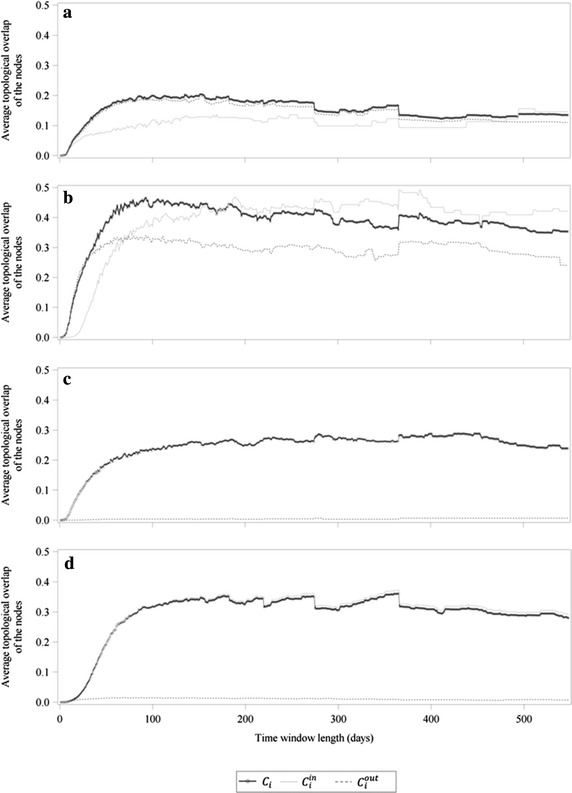
Mean average topological overlap of the nodes. Mean average undirected, ingoing and outgoing topological overlap of the nodes (*C*_*i*_, $C_{i}^{in}$ and $C_{i}^{out}$) separated by farm type, i.e. multipliers (**a**), farrowing farms (**b**), finishing farms (**c**) and farrow-to-finishing farms (**d**)

Figure 5 shows the mean average topological overlap of the nodes (*C*_*i*_, $$C_{i}^{in}$$ and $$C_{i}^{out} )$$ separated by the farm types multipliers (Fig. 5a), farrowing farms (Fig. 5b), finishing farms (Fig. 5c) and farrow-to-finishing farms (Fig. 5d). The finishing farms and the farrow-to-finishing farms showed for the mean *C*_*i*_ and $$C_{i}^{in}$$ relatively similar values, whereas the mean values of $$C_{i}^{out}$$ equalled for almost all time window lengths 0. Contrarily, multipliers and farrowing farms had a more differentiated course of *C*_*i*_, $$C_{i}^{in}$$ and $$C_{i}^{out}$$. For multipliers the mean values of $$C_{i}^{out}$$ showed quite similar values compared to *C*_*i*_, whereas the mean values of $$C_{i}^{in}$$ were slightly lower than the values of *C*_*i*_ and $$C_{i}^{out}$$ until a time window length of about 500 days, where $$C_{i}^{out}$$ became larger than the other two measurements. For farrowing farms the course of $$C_{i}^{in}$$ showed a smaller increase than $$C_{i}$$ until a time window length of 180 days where $$C_{i}^{in}$$ became larger than *C*_*i*_. $$C_{i}^{out}$$ had a quite similar course than *C*_*i*_ but moved down by about 0.10–0.15. Table 1 illustrates the mean average topological overlap of the nodes (*C*_*i*_, $$C_{i}^{in}$$ and $$C_{i}^{out} )$$ separated by the farm types for the time window lengths of 62 and 88 days and gives therefore a detailed section of the values illustrated in Fig. 5 for the time window length with the maximum values for *C*, *C*^*in*^ and *C*^*out*^.

**Table 1:** Mean (range) average topological overlap of the nodes

Farm type	n	Mean (range) average topological overlap of the nodes		
		Undirected (*C*_*i*_)	Ingoing ($C_{i}^{in}$)	Outgoing ($C_{i}^{out}$)
*Time window length of 62* *days*				
Multiplier	29	0.18 (0–0.8)	0.09 (0–1)	0.17 (0–0.87)
Farrowing farm	34	0.44 (0–0.86)	0.32 (0–1)	0.33 (0–0.93)
Finishing farm	153	0.21 (0–1)	0.21 (0–1)	0 (0–0.56)
Farrow-to-finishing farm	267	0.26 (0–1)	0.26 (0–1)	0.01 (0–0.97)
*Time window length of 88* *days*				
Multiplier	29	0.19 (0–0.82)	0.1 (0–1)	0.19 (0–0.82)
Farrowing farm	34	0.45 (0–0.89)	0.37 (0–1)	0.33 (0–0.91)
Finishing farm	153	0.23 (0–1)	0.23 (0–1)	0 (0–0.55)
Farrow-to-finishing farm	267	0.31 (0–1)	0.31 (0–1)	0.02 (0–0.96)

Mean (range) average undirected, ingoing and outgoing topological overlap of the nodes (*C*_*i*_, $C_{i}^{in}$ and $C_{i}^{out}$) separated by farm type for time window lengths of 62 and 88 days (corresponding to the maximum values of the temporal correlation coefficients *C*, *C* ^*in*^ and *C* ^*out*^)

**Fig. 6:**
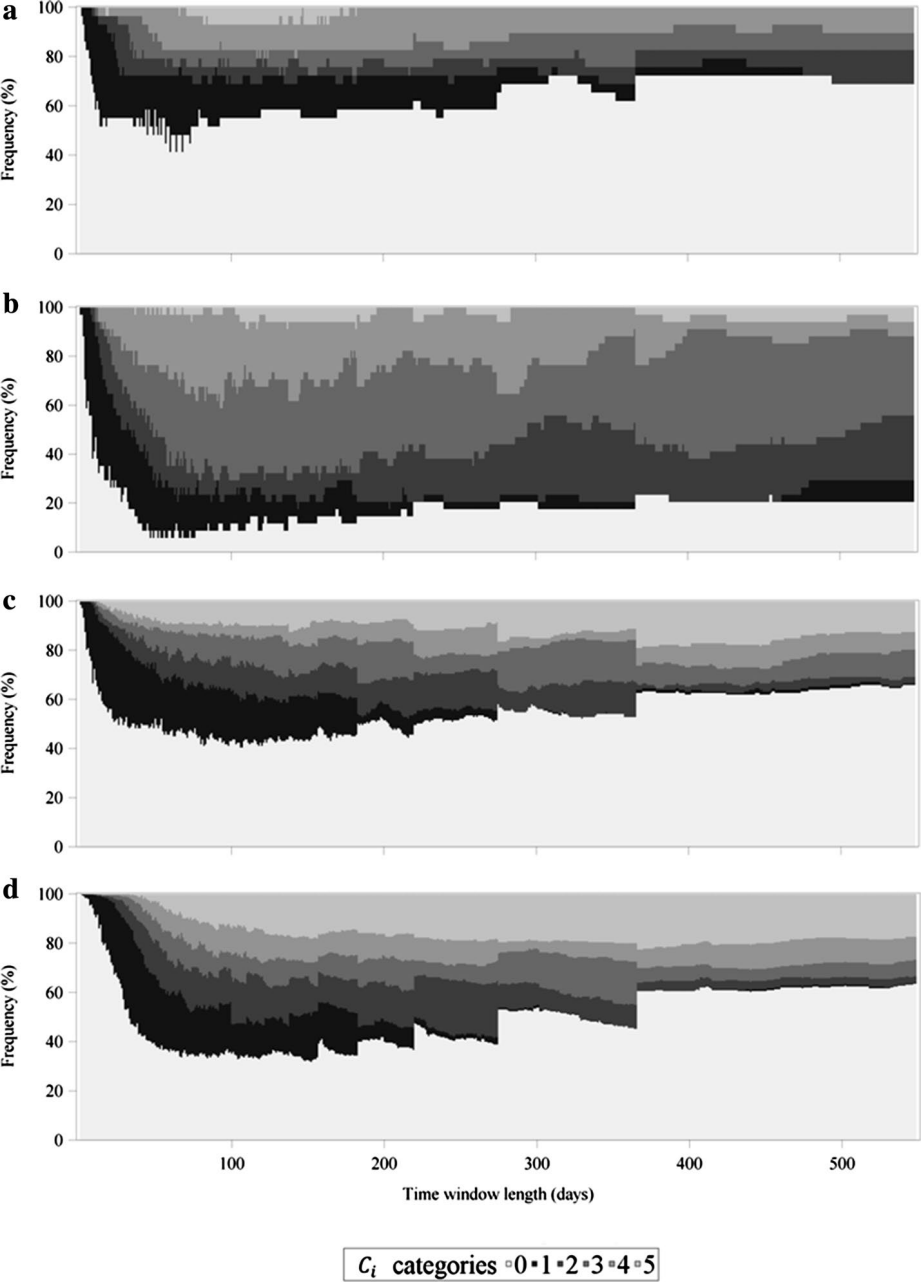
Frequency distribution of the undirected average topological overlap of the nodes (*C*_*i*_). The illustration is separated by farm type, i.e. multipliers (**a**), farrowing farms (**b**), finishing farms (**c**) and farrow-to-finishing farms (**d**). The categories are organized as follows: 0: *C*_*i*_ = 0, 1: 0 < *C*_*i*_ ≤ 0.2, 2: 0.2 < *C*_*i*_ ≤ 0.4, 3: 0.4 < *C*_*i*_ ≤ 0.6, 4: 0.6 < *C*_*i*_ ≤ 0.8, 5: 0.8 < *C*_*i*_ ≤ 1

**Fig. 7:**
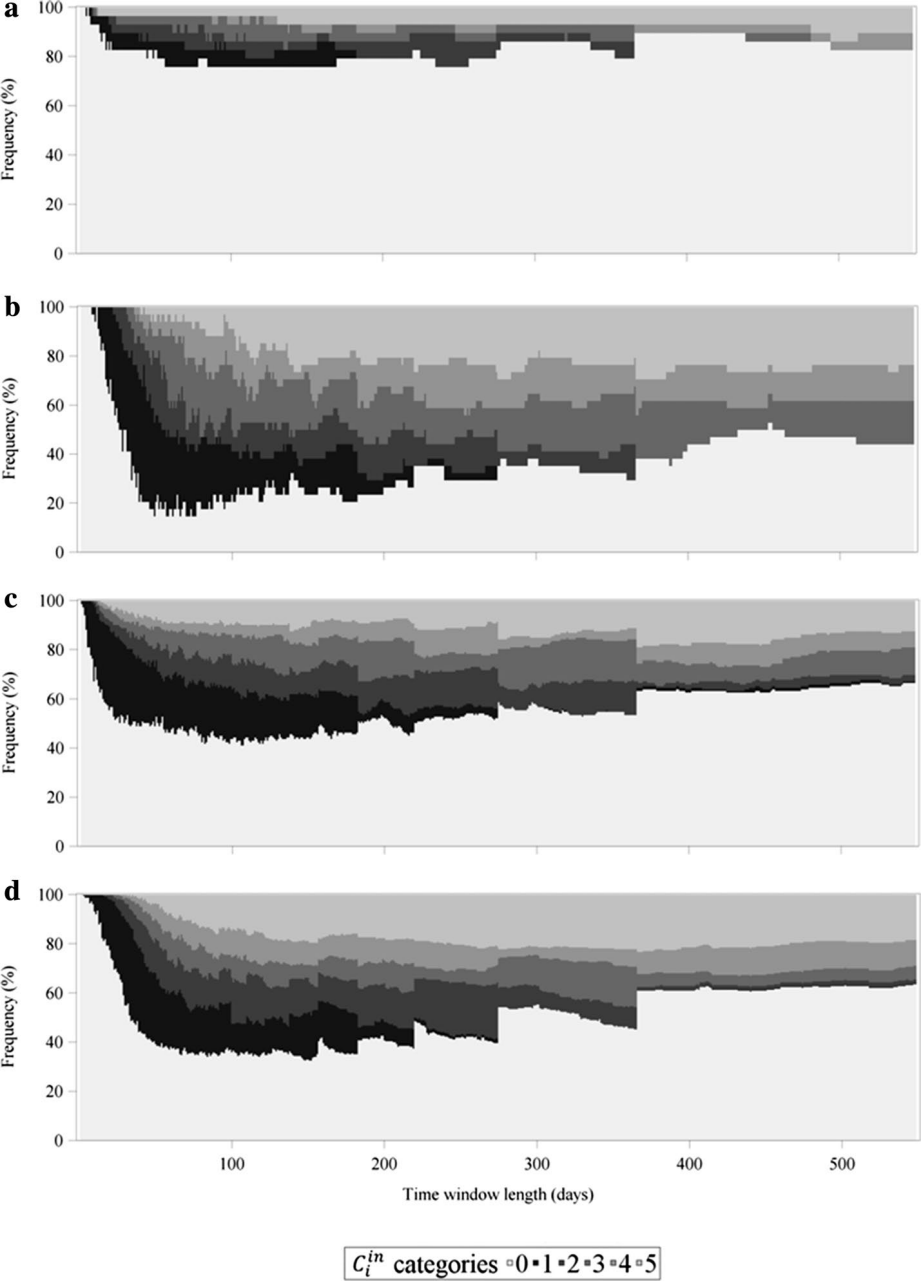
Frequency distribution of the ingoing average topological overlap of the nodes ($C_{i}^{in}$). The illustration is separated by farm type, i.e. multipliers (**a**), farrowing farms (**b**), finishing farms (**c**) and farrow-to-finishing farms (**d**). The categories are organized as follows: 0: $C_{i}^{in} = 0$, 1: $0 < C_{i}^{in} \le 0.2$, 2: $0.2 < C_{i}^{in} \le 0.4$, 3: $0.4 < C_{i}^{in} \le 0.6$, 4: $0.6 < C_{i}^{in} \le 0.8$, 5: $0.8 < C_{i}^{in} \le 1.$

**Fig. 8:**
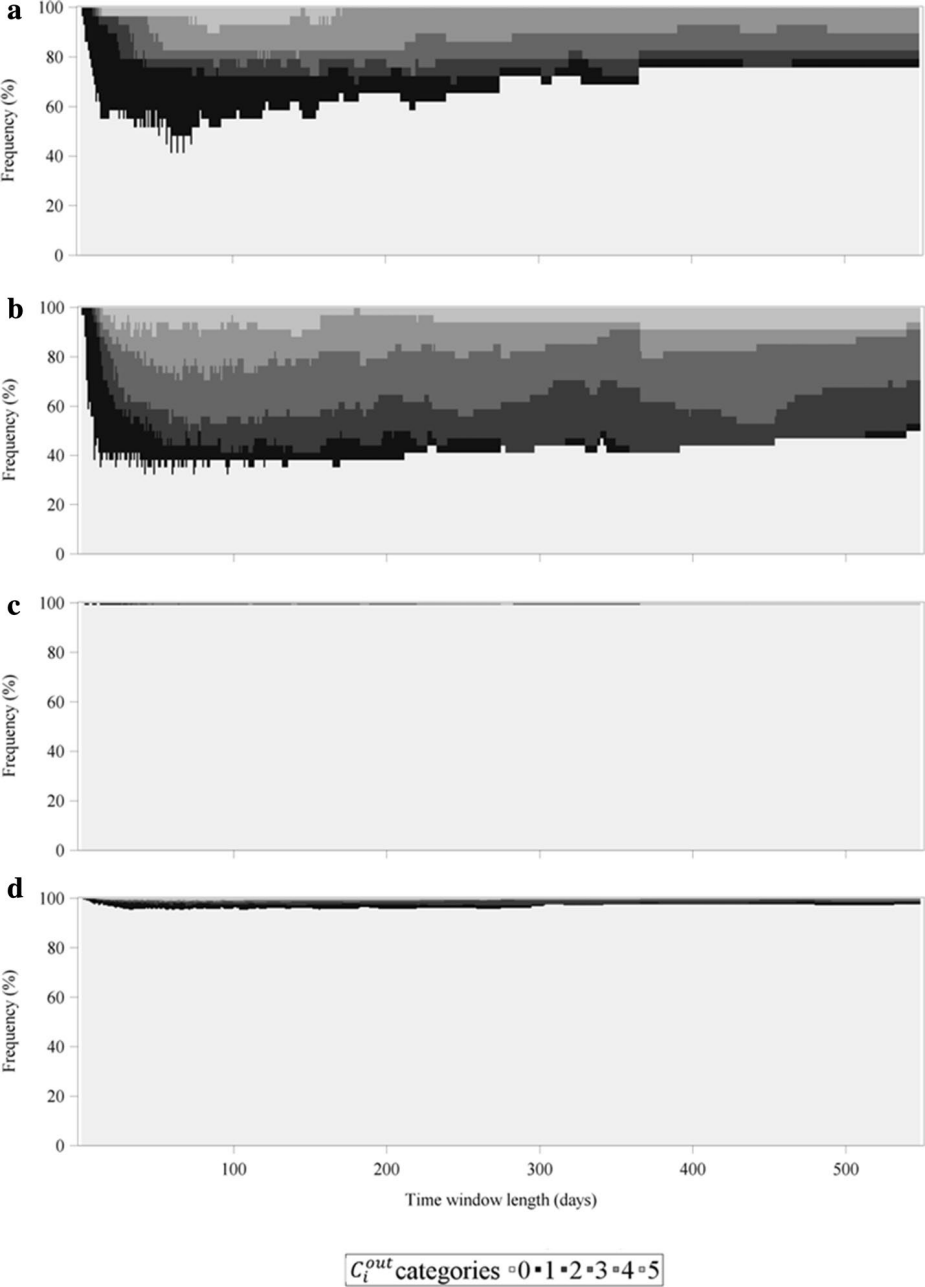
Frequency distribution of the outgoing average topological overlap of the nodes ($C_{i}^{out}$). The illustration is separated by farm type, i.e. multipliers (**a**), farrowing farms (**b**), finishing farms (**c**) and farrow-to-finishing farms (**d**). The categories are organized as follows: 0: $C_{i}^{out} = 0$, 1: $0 < C_{i}^{out} \le 0.2$, 2: $0.2 < C_{i}^{out} \le 0.4$, 3: $0.4 < C_{i}^{out} \le 0.6$, 4: $0.6 < C_{i}^{out} \le 0.8$, 5: $0.8 < C_{i}^{out} \le 1$

To get a better insight in the frequency distribution of *C*_*i*_, $C_{i}^{in}$ and $C_{i}^{out}$ depending on the chosen time window length and farm type, the values of *C*_*i*_, $C_{i}^{in}$ and $C_{i}^{out}$ were grouped into 6 different categories as illustrated in Figs. 6, 7 and 8. The first visual inspection reveals that, regarding *C*_*i*_ (Fig. 6) and $C_{i}^{out}$ (Fig. 8), the distribution of the categories for multipliers and farrowing farms were very similar, whereas they differed strongly when these two measures are compared for finishing farms and farrow-to-finishing farms. In contrast to this, the frequency distribution of *C*_*i*_ (Fig. 6) and $C_{i}^{in}$ (Fig. 7) was nearly identical for finishing farms and farrow-to-finishing farms and at least similar for multipliers and farrowing farms. To specify some particular observations, Fig. 6b illustrating the undirected case of the farrowing farms held the smallest percentage of *C*_*i*_ values equal to 0 (category 0), not only in comparison between the farm types but also compared to the frequency distribution diagrams related to the directed case (Figs. 7b, 8b). Furthermore, farrowing farms showed a higher percentage in the categories 4 and 5 in the undirected case compared to the other farm types. In contrast to that, finishing farms and farrow-to-finishing farms had, despite category 0, the majority of the farms in category 6 with the highest *C*_*i*_ values. Generally, the ratios between the higher categories differed between undirected, ingoing and outgoing cases. The frequency distribution of *C*_*i*_, $C_{i}^{in}$ and $C_{i}^{out}$ for the multipliers exemplarily illustrates that, in the undirected and the outgoing cases (Figs. 6a, 8a), category 5 occupied a larger area than category 6, but the frequency distribution regarding $C_{i}^{in}$ (Fig. 7a) showed a larger area of category 6 than category 5. Looking at the union of nonzero values for temporal correlation coefficients of the nodes in the ingoing case, the percentage of accumulated categories 1–6, i.e. $C_{i}^{in} > 0$, was largest for time window lengths between 70 and 110 days independent of the chosen farm type, whereby the course was not that pronounced for multipliers (Fig. 7a) compared to other farm types (Fig. 7b–d). From Fig. 8 it becomes obvious that almost exclusively the farm types at the beginning of the pork supply chain obtained values of $C_{i}^{out} > 0$. In contrast to the distribution of $C_{i}^{in}$, the largest percentages of categories 1–6 for $C_{i}^{out}$ could be observed for the time window lengths of 50–80 days.

## Discussion

### Convergence behaviour of the undirected, ingoing and outgoing temporal correlation coefficient (*C*, *C*^*in*^ and *C*^*out*^)

Due to the fact that the topological overlap represents the probability of an edge to persist between two consecutive snapshots and the temporal correlation coefficient is the average over all topological overlap values, both have to range between 0 and 1. In Fig. 3, the convergence behaviour of all three calculation methods (undirected, ingoing and outgoing) is illustrated for the example network of Fig. 1. All three calculation methods show the expected convergence against 1 with increasing number of added identical snapshots. The only difference for this example network can be seen between the undirected and the directed case. Here, the undirected temporal correlation coefficient showed higher values compared to the directed cases. This can be explained by the fact that in the undirected case the edge direction is ignored and therefore the probability for an edge to persist between two consecutive snapshots doubles which lead to higher values.

### Temporal correlation coefficient (*C*, *C*^*in*^ and *C*^*out*^) and average topological overlap of the nodes (*C*_*i*_, $C_{i}^{in}$ and $C_{i}^{out}$) calculated for a pig trade network

The results of the temporal correlation coefficient showed for all three cases (*C*, *C*^*in*^ and *C*^*out*^) that the choice of the time window length has an important impact on the edge persistence. *C*^*out*^ reached its maximum at a time window length of 62 days, indicating that most of the outgoing trade contacts had periodic patterns on average every 2 months. In contrast, the maximum of both *C* and *C*^*in*^ was reached at a time window length of 88 days. This corresponds to periodic patterns of on average 3 months. However, Lentz et al. (2013) showed periodical patterns of 180 days, representing the characteristic time scale of a pork supply chain. This result is two to three times higher than the findings of the present study. One explanation for these differences is that in the present study the abattoirs were excluded from the network analysis due to their dead-end characteristic for the transport of live animals. This also implies that a lot of farms, mainly the finishing and farrow-to-finishing farms, had no outgoing edges. This might be the main reason for the fact that *C* and *C*^*in*^ showed a similar course, whereas *C*^*out*^ could clearly be distinguished from them. Another point of consideration is that the fattening period including the trade contacts towards the abattoir, were also excluded from the network. This shortened the optimal time window length to 88 days for *C* and *C*^*in*^, indicated by the maximum values of the temporal correlation coefficients. The optimal time window length of 62 days for *C*^*out*^ can be explained by the relative constant relationships between multipliers and farrowing or farrow-to-finishing farms, respectively. They mainly deliver or receive, respectively, gilts for replacement. Additionally, there are also constant trade relations between farrowing farms and finishing farms every 2 months if the finishing farms do not follow the all-in-all-out-procedure, but fatten their animals compartment specific. To protect their health status within the herds it is recommended to keep the number of suppliers as low as possible in order to reduce the possibility of a disease entry (Waldmann and Wendt 2004). Therefore, the outgoing trade contacts of the multipliers and the farrowing farms have relatively constant purchasers. Additionally, multipliers or farrowing farms got a relatively high mean $C_{i}^{out}$, whereas the mean $C_{i}^{out}$ for the other farm types almost always equalled 0 (Fig. 5). However, these farm types only represent 13 % of the whole trade network. Due to the fact that for the calculation of the temporal correlation coefficients the edge configuration of all farms was taken into consideration, the values for *C*^*out*^ were lower than for *C* and *C*^*in*^.

The results of the average topological overlap of the nodes (Fig. 5; Table 1) indicated that the different farm types showed clearly different courses of *C*_*i*_, $C_{i}^{in}$ and $C_{i}^{out}$, depending on the position they occupy in the pork supply chain. Especially the farm types at the beginning of the pork supply chain, i.e. multipliers and farrowing farms had higher values of $C_{i}^{out}$ compared to finishing farms and farrow-to-finishing farms. The frequency distributions are another way to illustrate the differences in farm types regarding their edge configurations via ingoing and outgoing temporal correlation coefficients of the nodes. They provide details on the orders of magnitude in addition to the means of *C*_*i*_, $C_{i}^{in}$ and $C_{i}^{out}$ (Figs. 6, 7, 8). Between the distributions of the categories of *C*_*i*_ and $C_{i}^{out}$, similarities for multipliers and farrowing farms but huge differences for finishing farms and farrow-to-finishing farms could be observed. The small amount of nonzero $C_{i}^{out}$ values for finishing farms and farrow-to-finishing farms, i.e. the farm types at the end of the pork supply chain, can be explained by the exclusion of the abattoirs from the network analysis. Thus, these two farm types had almost no outgoing edges. To be more precise, only three finishing farms at all had outgoing edges. But these three farms could be classified as pure rearing farms which receive weaned piglets from a farrowing farm and delivered the piglets with about 30 kg body weight to another finishing farm. Therefore, they build a bridge between farrowing farms and the actual finishing farms with relatively stable trade contacts. However, all other finishing farms had no outgoing edges due to the exclusion of the abattoir. For a node without outgoing edges $C_{i}^{out}$ cannot be computed and is therefore set to zero.

The distribution of the categories of *C*_*i*_ and $C_{i}^{in}$ was nearly identical for finishing farms and farrow-to-finishing farms. In this case, no prominent differences for multipliers and farrowing farms could be observed. This indicates that the multipliers and the farrowing farms had ingoing edges with periodical patterns. Especially the farrowing farms received in constant time intervals gilts from the multipliers for their piglet production.

Looking at the frequency distributions of *C*_*i*_, $C_{i}^{in}$ and $C_{i}^{out}$ of the multipliers—as an example for the differing ratios in categories 1–6 between these measures—reveals a shift towards category 6 at the expense of mainly category 5 from *C*_*i*_, $C_{i}^{out}$ compared to $C_{i}^{in}$ (Figs. 6a, 7a, 8a). This can be explained by the fact that for the investigated pork supply chain most of the multipliers operated as hybrid breeding farms, whereas only a small amount could be assigned to nucleus breeding farms. The hybrid breeding farms received animals from the nucleus breeding farms in relatively constant intervals explaining the shift towards the higher amounts of $C_{i}^{in}$ values to category 6.

Despite the differences between the undirected and the directed cases, all figures (Figs. 6, 7, 8) illustrate that category 0 was the most represented. The only exception is Fig. 6b representing the categorized values of *C*_*i*_ for the farrowing farms. In the presented pork supply chain, this farm type occupied a middle position between the multipliers on the one end and the finishing and farrow-to-finishing farms at the other end. This is the reason that for both $C_{i}^{in}$ and $C_{i}^{out}$, and therefore also for *C*_*i*_ relatively high values for this farm type could be obtained.

### Importance of the distinction between ingoing and outgoing movements

For the edge configuration of the pork supply chain under investigation a clear differentiation between the ingoing and outgoing movements could be observed. Especially the different farm types showed specific courses of *C*, *C*^*in*^ and *C*^*out*^ as well as for *C*_*i*_, $C_{i}^{in}$ and $C_{i}^{out}$ depending on their position in the pork supply chain. These findings were confirmed by former studies of Büttner et al. (2013, 2015), where the centrality parameters also depend on the considered farm type. Especially the farm types at the beginning of the pork supply chain, i.e. multipliers and farrowing farms had significant higher values for the centrality parameters regarding the outgoing trade contacts compared to the farm types at the end of the pork supply chain, i.e. finishing and farrow-to-finishing farms. Furthermore, these studies showed that the rankings of the centrality parameters based on outgoing edges (e.g. out-degree, outgoing infection chain) had a more stable ranking than the centrality parameters based on the ingoing trade contacts (e.g. in-degree, ingoing infection chain). These findings gave evidence to the fact that there were some highly central farms delivering animals to the majority of this network. This results in a high value for the centrality parameters based on the outgoing trade contacts. However, it has to be considered that the values of the centrality parameters cannot directly be compared to the results of the temporal correlation coefficient. The reason for this is the fact that during the calculation of the centrality parameters only one node is focussed including the attached edges but it is unimportant which node stands on the other side of this edge. In contrast to this, during the calculation of the temporal correlation coefficient these pairs of nodes and especially the connection between two specific nodes are considered. Thus, an additional statement on stability of the connections between the nodes is made. This has to be kept in mind when talking about temporal correlation coefficients. Despite the differences between the calculation of centrality parameters and the temporal correlation coefficient both can pass on more information if the direction of the edges is considered. The direction of the movements is of special importance for the analysis of disease spread within such directed networks. Here, a high number of ingoing movements increases the probability to get an infection, whereas a high number of outgoing movements means that the probability for spreading an infection increases. For the investigation of aggregated networks it is therefore very important to check the quality of this aggregation, in terms of high values for the temporal correlation coefficient. Furthermore, in order to keep the information of the directed network it is important to calculate not only the undirected temporal correlation coefficient *C*_*i*_ but also its directed counterparts $C_{i}^{in}$ and $C_{i}^{out}$ as more specific quality measures.

## Conclusions

The analysis of temporal networks as well as the investigation of their structural characteristics are still in their infancies. Thus, appropriate methods which help to examine how the structure of temporal networks may affect the dynamics of processes occurring on it, e.g. disease transmission, are still missing. Additionally, most of the newly described methods are initially based on undirected networks. However, especially if the direction of the edges is a main characteristic of the network, such as for the pig trade network, neglecting this property leads to an essential loss of information. Thus, the present paper provides methodologies to maintain this information by calculating the directed temporal correlation coefficients $C_{i}^{in}$ and $C_{i}^{out}$ representing an adaption of the temporal correlation coefficient to directed networks. Furthermore, it could be shown that depending on the position in the pork supply chain the edge configuration of specific farm types differentiated.

To conclude, considering the yet known dependencies and issues in dealing with the analysis of temporal network analysis, the temporal correlation coefficient is a valuable tool to understand the structural dynamics of these systems, as it assesses the consistency of the edge configuration. The adaption of this measure for directed networks may help to preserve meaningful additional information about the investigated network that might get lost, if the edge directions are ignored.

## Additional file

10.1186/s40064-016-2875-0 Detailed calculation steps of the undirected, the ingoing and the outgoing temporal correlation coefficient (C, C^in^, C^out^).
