# Supplementary material for: Temporal correlation coefficient for directed networks
Source: Springerplus. 2016 Jul 28;5(1):1198. doi: 10.1186/s40064-016-2875-0 (PMC4963342; doi:10.1186/s40064-016-2875-0)
Supplement: Supplementary file 1 — 10.1186/s40064-016-2875-0 Detailed calculation steps of the undirected, the ingoing and the outgoing temporal correlation coefficient (C, Cin, Cout). [file 40064_2016_2875_MOESM1_ESM.docx]

**Temporal correlation coefficient for directed networks – Supplementary material**

Kathrin Büttner^*^, Jennifer Salau, Joachim Krieter

Institute of Animal Breeding and Husbandry, Christian-Albrechts-University, Olshausenstr. 40, D-24098 Kiel, Germany

^*^ Corresponding author: Kathrin Büttner

Tel.: +49 431 880 4537

Fax: +49 431 880 2588

E-mail address: [kbuettner@tierzucht.uni-kiel.de](mailto:kbuettner@tierzucht.uni-kiel.de)

In this supplementary material we describe the single calculation steps for the temporal correlation coefficient in the undirected, the ingoing and the outgoing case with the help of a small example network (Figure S1). Figure S1 depicts an example network consisting of 4 temporal snapshots with directional information given by the arrow tips of the edges.

| **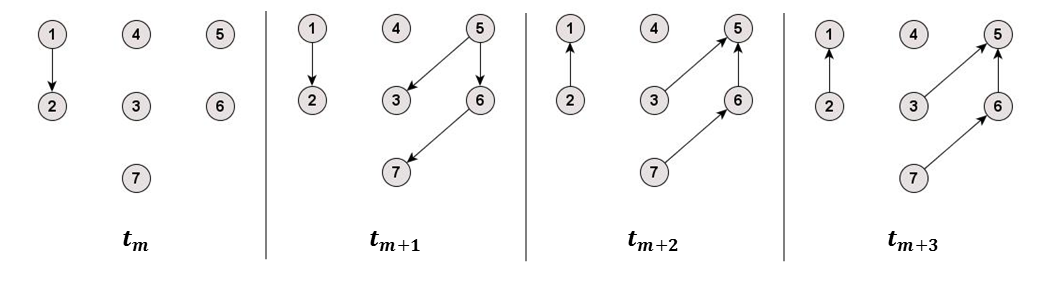** |
| --- |

**Figure S1:** Example network of 4 different temporal snapshots ($t_{m}, \ldots, t_{m+3}$).

**1. Undirected temporal correlation coefficient** $\boldsymbol{C}$

The single calculation steps of the undirected temporal correlation coefficient $C$ are explained in the following. To achieve $C$ the edge directions in Figure S1 are neglected, i.e. the arrow pointing from node one to node two in the first snapshot and the arrow pointing from node two to node one in the third snapshot are treated equally.

***1^st^ step: Calculation of*** $\boldsymbol{C}_{\boldsymbol{i}}\left( \boldsymbol{t}_{\boldsymbol{m}}\boldsymbol{,}\boldsymbol{t}_{\boldsymbol{m+1}} \right)$***:***

| $C_{i}\left( t_{m}, t_{m+1} \right)$*:* | $C_{i}\left( t_{m+1}, t_{m+2} \right)$*:* | $C_{i}\left( t_{m+2}, t_{m+3} \right)$*:* |
| --- | --- | --- |
| $C_{i=1}\left( t_{m}, t_{m+1} \right)=1$ | $C_{i=1}\left( t_{m+1}, t_{m+2} \right)=1$ | $C_{i=1}\left( t_{m+2}, t_{m+3} \right)=1$ |
| $C_{i=2}\left( t_{m}, t_{m+1} \right)=1$ | $C_{i=2}\left( t_{m+1}, t_{m+2} \right)=1$ | $C_{i=2}\left( t_{m+2}, t_{m+3} \right)=1$ |
| $C_{i=3}\left( t_{m}, t_{m+1} \right)=0$ | $C_{i=3}\left( t_{m+1}, t_{m+2} \right)=1$ | $C_{i=3}\left( t_{m+2}, t_{m+3} \right)=1$ |
| $C_{i=4}\left( t_{m}, t_{m+1} \right)=0$ | $C_{i=4}\left( t_{m+1}, t_{m+2} \right)=0$ | $C_{i=4}\left( t_{m+2}, t_{m+3} \right)=0$ |
| $C_{i=5}\left( t_{m}, t_{m+1} \right)=0$ | $C_{i=5}\left( t_{m+1}, t_{m+2} \right)=1$ | $C_{i=5}\left( t_{m+2}, t_{m+3} \right)=1$ |
| $C_{i=6}\left( t_{m}, t_{m+1} \right)=0$ | $C_{i=6}\left( t_{m+1}, t_{m+2} \right)=1$ | $C_{i=6}\left( t_{m+2}, t_{m+3} \right)=1$ |
| $C_{i=7}\left( t_{m}, t_{m+1} \right)=0$ | $C_{i=7}\left( t_{m+1}, t_{m+2} \right)=1$ | $C_{i=7}\left( t_{m+2}, t_{m+3} \right)=1$ |

***2^nd^ step: Calculation of*** $\boldsymbol{C}_{\boldsymbol{m}}$***:***

$C_{m}= \frac{1}{max[A\left( t_{m} \right), A\left( t_{m+1} \right)]}\sum_{i=1}^{N} C_{i}(t_{m}, t_{m+1})\approx0.33$

$C_{m+1}= \frac{1}{max[A\left( t_{m+1} \right), A\left( t_{m+2} \right)]}\sum_{i=1}^{N} C_{i}(t_{m+1}, t_{m+2})=1$

$C_{m+2}= \frac{1}{max[A\left( t_{m+2} \right), A\left( t_{m+3} \right)]}\sum_{i=1}^{N} C_{i}(t_{m+2}, t_{m+3})= 1$

***3^rd^ step: Calculation of*** $\boldsymbol{C}$***:***

$C= \frac{1}{M-1}\sum_{m}^{M-1} C_{m}\approx0.78$

**2. Ingoing temporal correlation coefficient** $\boldsymbol{C}^{\boldsymbol{in}}$

In the following the single calculation steps for the ingoing temporal correlation coefficient $C^{in}$ for the example network (Figure S1) are illustrated:

***1^st^ step: Calculation of*** $\boldsymbol{C}_{\boldsymbol{i}}^{\boldsymbol{in}}\left( \boldsymbol{t}_{\boldsymbol{m}}\boldsymbol{,}\boldsymbol{t}_{\boldsymbol{m+1}} \right)$***:***

| $C_{i}^{in}\left( t_{m}, t_{m+1} \right)$*:* | $C_{i}^{in}\left( t_{m+1}, t_{m+2} \right)$*:* | $C_{i}^{in}\left( t_{m+2}, t_{m+3} \right)$*:* |
| --- | --- | --- |
| $C_{i=1}^{in}\left( t_{m}, t_{m+1} \right)=0$ | $C_{i=1}^{in}\left( t_{m+1}, t_{m+2} \right)=0$ | $C_{i=1}^{in}\left( t_{m+2}, t_{m+3} \right)=1$ |
| $C_{i=2}^{in}\left( t_{m}, t_{m+1} \right)=1$ | $C_{i=2}^{in}\left( t_{m+1}, t_{m+2} \right)=0$ | $C_{i=2}^{in}\left( t_{m+2}, t_{m+3} \right)=0$ |
| $C_{i=3}^{in}\left( t_{m}, t_{m+1} \right)=0$ | $C_{i=3}^{in}\left( t_{m+1}, t_{m+2} \right)=0$ | $C_{i=3}^{in}\left( t_{m+2}, t_{m+3} \right)=0$ |
| $C_{i=4}^{in}\left( t_{m}, t_{m+1} \right)=0$ | $C_{i=4}^{in}\left( t_{m+1}, t_{m+2} \right)=0$ | $C_{i=4}^{in}\left( t_{m+2}, t_{m+3} \right)=0$ |
| $C_{i=5}^{in}\left( t_{m}, t_{m+1} \right)=0$ | $C_{i=5}^{in}\left( t_{m+1}, t_{m+2} \right)=0$ | $C_{i=5}^{in}\left( t_{m+2}, t_{m+3} \right)=1$ |
| $C_{i=6}^{in}\left( t_{m}, t_{m+1} \right)=0$ | $C_{i=6}^{in}\left( t_{m+1}, t_{m+2} \right)=0$ | $C_{i=6}^{in}\left( t_{m+2}, t_{m+3} \right)=1$ |
| $C_{i=7}^{in}\left( t_{m}, t_{m+1} \right)=0$ | $C_{i=7}^{in}\left( t_{m+1}, t_{m+2} \right)=0$ | $C_{i=7}^{in}\left( t_{m+2}, t_{m+3} \right)=0$ |

***2^nd^ step: Calculation of*** $\boldsymbol{C}_{\boldsymbol{m}}^{\boldsymbol{in}}$***:***

$C_{m}^{in}= \frac{1}{max[A^{in}\left( t_{m} \right), A^{in}\left( t_{m+1} \right)]}\sum_{i=1}^{N} C_{i}^{in}(t_{m}, t_{m+1})= 0.25$

$C_{m+1}^{in}= \frac{1}{max[A^{in}\left( t_{m+1} \right), A^{in}\left( t_{m+2} \right)]}\sum_{i=1}^{N} C_{i}^{in}(t_{m+1}, t_{m+2})= 0$

$C_{m+2}^{in}= \frac{1}{max[A^{in}\left( t_{m+2} \right), A^{in}\left( t_{m+3} \right)]}\sum_{i=1}^{N} C_{i}^{in}(t_{m+2}, t_{m+3})= 1$

***3^rd^ step: Calculation of*** $\boldsymbol{C}^{\boldsymbol{in}}$***:***

$C^{in}= \frac{1}{M-1}\sum_{m}^{M-1} C_{m}^{in}\approx0.416$

**3. Outgoing temporal correlation coefficient** $\boldsymbol{C}^{\boldsymbol{out}}$

In the following the single calculation steps for the outgoing temporal correlation coefficient $C^{out}$ for the example network (Figure S1) are illustrated:

***1^st^ step: Calculation of*** $\boldsymbol{C}_{\boldsymbol{i}}^{\boldsymbol{out}}\left( \boldsymbol{t}_{\boldsymbol{m}}\boldsymbol{,}\boldsymbol{t}_{\boldsymbol{m+1}} \right)$***:***

| $C_{i}^{out}\left( t_{m}, t_{m+1} \right)$*:* | $C_{i}^{out}\left( t_{m+1}, t_{m+2} \right)$*:* | $C_{i}^{out}\left( t_{m+2}, t_{m+3} \right)$*:* |
| --- | --- | --- |
| $C_{i=1}^{out}\left( t_{m}, t_{m+1} \right)=1$ | $C_{i=1}^{out}\left( t_{m+1}, t_{m+2} \right)=0$ | $C_{i=1}^{out}\left( t_{m+2}, t_{m+3} \right)=0$ |
| $C_{i=2}^{out}\left( t_{m}, t_{m+1} \right)=0$ | $C_{i=2}^{out}\left( t_{m+1}, t_{m+2} \right)=0$ | $C_{i=2}^{out}\left( t_{m+2}, t_{m+3} \right)=1$ |
| $C_{i=3}^{out}\left( t_{m}, t_{m+1} \right)=0$ | $C_{i=3}^{out}\left( t_{m+1}, t_{m+2} \right)=0$ | $C_{i=3}^{out}\left( t_{m+2}, t_{m+3} \right)=1$ |
| $C_{i=4}^{out}\left( t_{m}, t_{m+1} \right)=0$ | $C_{i=4}^{out}\left( t_{m+1}, t_{m+2} \right)=0$ | $C_{i=4}^{out}\left( t_{m+2}, t_{m+3} \right)=0$ |
| $C_{i=5}^{out}\left( t_{m}, t_{m+1} \right)=0$ | $C_{i=5}^{out}\left( t_{m+1}, t_{m+2} \right)=0$ | $C_{i=5}^{out}\left( t_{m+2}, t_{m+3} \right)=0$ |
| $C_{i=6}^{out}\left( t_{m}, t_{m+1} \right)=0$ | $C_{i=6}^{out}\left( t_{m+1}, t_{m+2} \right)=0$ | $C_{i=6}^{out}\left( t_{m+2}, t_{m+3} \right)=1$ |
| $C_{i=7}^{out}\left( t_{m}, t_{m+1} \right)=0$ | $C_{i=7}^{out}\left( t_{m+1}, t_{m+2} \right)=0$ | $C_{i=7}^{out}\left( t_{m+2}, t_{m+3} \right)=1$ |

***2^nd^ step: Calculation of*** $\boldsymbol{C}_{\boldsymbol{m}}^{\boldsymbol{out}}$

$C_{m}^{out}= \frac{1}{max[A^{out}\left( t_{m} \right), A^{out}\left( t_{m+1} \right)]}\sum_{i=1}^{N} C_{i}^{out}(t_{m}, t_{m+1})\approx0.33$

$C_{m+1}^{out}= \frac{1}{max[A^{out}\left( t_{m+1} \right), A^{out}\left( t_{m+2} \right)]}\sum_{i=1}^{N} C_{i}^{out}(t_{m+1}, t_{m+2})=0$

$C_{m+2}^{out}= \frac{1}{max[A^{out}\left( t_{m+2} \right), A^{out}\left( t_{m+3} \right)]}\sum_{i=1}^{N} C_{i}^{out}(t_{m+2}, t_{m+3})= 1$

***3^rd^ step: Calculation of*** $\boldsymbol{C}^{\boldsymbol{out}}$***:***

$C^{out}= \frac{1}{M-1}\sum_{m}^{M-1} C_{m}^{out}\approx0.444$
